# Psychobiological Consequences of Childhood Sexual Abuse: Current Knowledge and Clinical Implications

**DOI:** 10.3389/fnins.2021.771511

**Published:** 2021-12-02

**Authors:** Luisa Lo Iacono, Cristina Trentini, Valeria Carola

**Affiliations:** ^1^Department of Dynamic and Clinical Psychology, and Health Studies, Sapienza University of Rome, Rome, Italy; ^2^IRCCS Santa Lucia Foundation, Rome, Italy

**Keywords:** childhood sexual abuse, clinical psychology, immune system, HPA axis, telomeres

## Abstract

A large body of research has documented the long-term harms of childhood sexual abuse (CSA) on an individual’s emotional-adaptive function and mental health. Recent studies have also provided evidence of the biological impact of CSA, implicating specific alterations in many systems, including the endocrine and immune systems, and in DNA and chromatin, in the pathogenesis of medical disorders. Although the effects of CSA are often examined with regard to the general impact of early-life traumatic experiences, the study of CSA per sè, as a trigger of specific pathogenic pathways, would be more appropriate to understand their long-term implications and develop tailored diagnostic and therapeutic strategies. Based on these premises, this narrative minireview summarizes the research on the short-term and long-term sequelae of CSA, focusing on dysregulation of the hypothalamic-pituitary-adrenal (HPA) axis, the effects on the immune system, and the changes to DNA through altered methylation. Also, we discuss the literature that examines dysfunctional DNA telomere erosion and oxidative stress markers as a sign of CSA. Finally, recent evidence of the intergenerational transmission of the effects of CSA is reported. The impact of CSA on brain connectivity and functions is out of the scope of this review, thus brain imaging studies are not included. The results of this minireview are discussed, considering their implications for prevention and clinical practice.

## Introduction

Childhood sexual abuse (CSA) is a severe public health concern, affecting roughly 1 in 4 girls and 1 in 13 boys worldwide (Center for Disease Control and Prevention, 2021).

As with other forms of abuse, CSA is characterized by the complex manipulation and coercion of the perpetrator and the unbalanced and power-based relationship that is established by leveraging and exploiting vulnerability.

Childhood sexual abuse includes sexually connotated physical contact or non-contact activities. The former includes intercourse, attempted intercourse, or oral-genital contact with the penis, fingers, or any object; masturbation; and fondling the genitals or other erogenous areas through the clothing or directly. The latter entails forcing a child to participate in adult sexual pleasure (such as sexual harassment and prostitution) or exposing a child to adult sexual activities, such as pornography, voyeurism, and exhibitionism (Putnam, 2003; Slep et al., 2015).

Childhood sexual abuse alters the normal developmental trajectories that are necessary for healthy socioemotional function (Langevin et al., 2016; Clayton et al., 2018), increasing the likelihood of a child experiencing sociorelational difficulties, cognitive dysfunction, depression, anxiety, internalization and externalization of problems, sexualized behaviors, and post-traumatic symptoms (Saywitz et al., 2000). These negative outcomes are exacerbated by the cumulative impact of several types of victimization, to which the child is commonly exposed in his or her family (Putnam et al., 2013; Ford and Delker, 2018; Goodman et al., 2020).

A large body of research has documented that the negative effects of CSA can persist until adulthood. Associations between CSA and a wide range of psychiatric outcomes, including post-traumatic stress disorder (PTSD), schizophrenia, conversion disorder, borderline personality disorder, eating disorders, anxiety, and depression, have been described (Hailes et al., 2019). Further, CSA has been linked to a greater risk for substance abuse (Halpern et al., 2018), suicidal ideation and suicide-related behavior (Afifi et al., 2014; Devries et al., 2014), and adult victimization (Aakvaag et al., 2017).

Notably, as in other forms of childhood maltreatment, children who are exposed to SA are likely to become abusive parents (Assink et al., 2018), supporting the existence of intergenerational transmission of abuse. Specifically, this phenomenon appears to be mediated by the construction of disorganized attachment in the child (Madigan et al., 2006; Cyr et al., 2010).

Beyond being at high risk for lifelong mental disturbances, individuals with CSA are also vulnerable to disruptions in physical health. These individuals often develop a wide variety of symptoms that are often medically unexplained, including chronic pain; sleep problems; adult-onset arthritis; fibromyalgia; long-term fatigue; diabetes; and circulatory, digestive, respiratory, musculoskeletal, reproductive, and neurological problems (Sigurdardottir and Halldorsdottir, 2018).

Several changes in physiological functions have been described as a consequence of CSA. Considering that such effects might be particularly relevant because they are involved in the modulation of CSA-induced psychological and physical disturbances, this minireview will summarize research on the short-term and long-term sequelae of CSA, focusing on the dysregulation of the hypothalamic-pituitary-adrenal (HPA) axis, the effects on the immune system, and the changes to DNA through altered methylation. Also, the literature on dysfunctional cellular processes, such as DNA telomere erosion and oxidative stress markers as a sign of CSA, will be presented. We will conclude with recent evidence on the pathways through which CSA might be transmitted to offspring.

## Methodology

We performed a literature search of studies on the effects of CSA within the following topics: biological impact of CSA; effects of CSA on the HPA axis, immune system, DNA methylation, and cellular processes in adults; and transgenerational inheritance of the impacts of CSA. The impact of CSA on brain connectivity and functions is out of the scope of this review, thus brain imaging studies are not included. The search was conducted primarily using PubMed Advanced Search Builder, as in the following example: “Child sexual abuse” AND (“HPA” OR “psychological outcomes” OR “inflammation” OR “telomere” OR “DNA methylation”) NOT (“Neglect” OR “Childhood Maltreatment”). Other keywords and mesh words included cortisol, cortisol awakening response, immune activity, inflammatory markers, peripheral biomarkers, blood biomarkers, epigenetics, genetics, telomere length, DNA, RNA, childhood, adolescence, rape, sexual violence, sexual victimization, and intergenerational transmission of trauma. Supplementary articles were included through performing a manual search, analyzing the references of the most relevant articles, and scanning all papers from meta-analyses and reviews. All selected papers were written in English, and most were published between 2015 and 2021.

## Results

### The Sequelae of Sexual Abuse in Children

The literature on the immediate biological effects of CSA on children is limited (Table 1). These studies usually aim to understand the acute response and the resulting compensatory physiological changes, which, by interacting with an individual’s genetic makeup, form the basis of long-term dysfunctions.

**TABLE 1:** Short-term biological consequences of childhood sexual abuse (CSA) exposure.

Article	Age	Blood/Saliva/Buccal	Sample size	Biological function affected	Main results	
Muller et al., 2014	7–12 years	Blood	11	HPA	**↓**	Children in institutional settings demonstrated an attenuated stress reactivity pattern, interpreted as an apparent lack of an appropriate HPA response when faced with a further stressor (e.g., forensic interview and medical examination).
Şimşek et al., 2016	9–17 years	Blood	38	HPA	**↑**	In children who developed a PTSD, oxidative stress was higher following multiple abuses and sexual abuses within the family.
Atabay and Arman, 2019	10–17 years	Blood	90	HPA	**↑**	CSA group showed high oxidative stress and low antioxidant process profile.
Muller et al., 2014	7–12 years	Blood	11	Immune system	**↑**	SA children in institutional setting had higher morning IL-6 concentrations during their clinic visit, which were inversely correlated with plasma cortisol concentrations.
Esteves et al., 2020	4, 12, 18 months	Buccal	155	Epigenetic and Chromatin	**↓**	Mothers’ high scores in the ACE questionnaire predicted shorter telomere length and emerging (mainly externalizing) behavioral problems in the offspring.
Shalev et al., 2013	5 and 10 years	Buccal	236	Epigenetic and Chromatin	**↓**	Accelerated telomere erosion was observed in children who were exposed to multiple forms of violence and the effects worsen over time.
Ridout et al., 2018	3–5 years	Saliva	250	Epigenetic and Chromatin	**↑**	Children who experienced moderate-severe levels of maltreatment within 6 months prior to the analysis, had longer telomeres and higher mtDNA, possibly reflecting compensatory changes in response to recent trauma.

*HPA, hypothalamic-pituitary-adrenal axis; IL-6, Interleukin 6; ACE, adverse childhood experiences; mtDNA, mitochondrial DNA.*

Specifically, studies in child and adolescent victims of sexual abuse focus primarily on the immediate impact on the HPA axis and immune system, through the analysis of blood cortisol and cytokines levels, respectively.

A large body of research has assessed the impact of CSA on cellular processes, assaying, for instance, the length of DNA telomeres as a measure of cellular aging and determining the levels of oxidative stress due to the cortisol response. Such facets are generally evaluated using an oxidant stress index (OSI), defined by combining measures of various blood parameters that are involved in antioxidation (such as levels of superoxide dismutase) and oxidation processes (including ROS and DNA damage). Few studies, as reported here, have assessed these variables at a young age.

#### Hypothalamic-Pituitary-Adrenal and Immune System Dysfunction in Child Victims of Childhood Sexual Abuse

Excessive or prolonged stress dysregulates the HPA axis. Şimşek et al. (2016) investigated whether morning serum cortisol levels (obtained between 10:00 and 12:00 AM) differ between children who developed PTSD or not following sexual abuse. Notably, cortisol levels were lower in children who reported PTSD and who were victims of multiple assaults, compared to children without PTSD and who were victims of a single assault. Moreover, in children with PTSD, cortisol levels decreased as time from the episode of abuse elapsed. According to the authors, the cortisol levels decrease in time may reflect an adaptive process aimed at preventing the negative impact of prolonged exposure to high cortisol levels on cerebral structures (particularly, on frontal cortex and hippocampus).

Muller et al. (2014) examined the HPA axis and immune dysregulation at the first visit of children to a sexual abuse clinic, measuring plasma cortisol and IL-6 concentrations immediately after the stressful forensic interview. Notably, the levels varied, depending on environmental differences – i.e., place of residence (familial home or institution): children who resided at home had higher cortisol concentrations and no detectable IL-6 in their plasma. In contrast, children who lived in institutions had lower cortisol concentrations and detectable levels of IL-6. This study suggests that sexually abused children who reside in institutional settings have attenuated stress reactivity patterns when faced with an additional stressor, such as a forensic interview or medical examination.

#### Dysfunctional Cellular Processes in Children Victims of Childhood Sexual Abuse

Prolonged cortisol exposure and a chronic inflammatory response can lead to oxidative stress and DNA damage at a young age. In this context, Atabay and Arman (2019) found that sexually abused children have significantly higher oxidative stress index (OSI) values compared with an age-/gender-matched control group.

Şimşek et al. (2016) reported that specific features of sexual abuse differentially affect the outcomes. In their study, oxidative stress was higher in victims of multiple abuses and sexual abuse within the family. Multiple assaults lowered the levels of the antioxidant coenzyme Q and superoxide dismutase.

One potential mechanism that links stress to cellular aging in humans is the erosion of DNA telomeres. Exposure to early-life traumatic events accelerates telomere erosion in childhood – an effect that worsens over time (Shalev et al., 2013). However, controversial data by Ridout et al. (2018) demonstrated longer telomeres in a sample of children who were exposed to moderate or severe maltreatment, including CSA, in the previous 6 months. The authors speculated that their data reflect altered telomerase activity. Telomerase can be activated in an attempt to overcome telomere attrition and protect against oxidative stress. However, further studies are needed to understand the effects of early stress on telomere erosion and determine whether CSA has a specific impact on this process.

### Long-Term Effects of Childhood Sexual Abuse

#### Hypothalamic-Pituitary-Adrenal Dysfunction in Adults With a History of Childhood Sexual Abuse

The early environment is a significant factor in the programming of the HPA axis in adults, and in turn, dysregulation of the HPA axis can mediate one’s vulnerability to stress-related psychiatric disorders (Table 2).

**TABLE 2:** Long-term biological consequences of childhood sexual abuse (CSA) exposure.

Article	Adult/Child	Age	Blood/Saliva/Buccal/Hair	Sample size	Biological function affected	Main results	
Heim et al., 2000	A	18–45 years	Blood	49	HPA	↑	Women with a history of childhood sexual/physical abuse exhibited increased pituitary adrenal and autonomic responses to stress compared to controls.
Schalinski et al., 2015	A	16–56 years	Saliva-Hair	43	HPA	↕	CSA group displayed lower levels of saliva cortisol, measured after exposure to idiographic adverse experiences (phasic), and higher hair cortisol levels compared to control (tonic).
Hulme et al., 2015	A	29–66 years	Saliva	17	HPA	↑	CSA is associated with long-lasting changes in diurnal patterns of cortisol secretion in bariatric surgery patients. Thus, altered HPA axis regulation may be risk factor to adult obesity in CSA victims.
Bublitz and Stroud, 2012	A	18–40 years	Saliva	135	HPA	↑	Women with CSA histories displayed significantly higher pre-pregnancy BMI, greater anxiety symptoms, increasing cortisol awakening response, and did not display the protective shift to responsiveness, over pregnancy.
Bertone-Johnson et al., 2012	A	25–42 years	Blood	702	Immune system	↑	CRP and IL-6 levels are higher in CSA survivors compared to the non-abused group, the association between sexual abuse and inflammation markers is slightly stronger when the abuse occurs in adolescence rather than childhood.
McCall et al., 2018	A	40–60 years	–	100	Immune system	↓	The rates of CSA in people living with HIV positivity are estimated at 30 to 53%, together with high rates of illicit drug use, and sex trade experiences.
LeGrand et al., 2015 (*review*)	A	–	–	–	Immune system	↓	Trauma is associated with lower rates of HIV disclosure and increases in risk behaviors, poorer mental health, substance abuse, and immunologic outcomes.
D’Elia et al., 2018 *(review)*	A	–	–	–	Immune system	↕	Articles based on children samples reported blurred results, while in adult samples the effects seem to be clearer with most studies reporting increased inflammatory activity, i.e., higher levels of IL-6, TNF-α, and C-reactive protein.
Grosse et al., 2016	A	18–65 years	Blood	394	Immune system	↑	In Major Depressive Disorder patients a linear relationship was shown between CSA and pro-inflammatory cytokine levels, while more recent stressful life events were not related to these inflammatory markers.
Cai et al., 2015	A	>18 years	Blood-Saliva	1,1647	Epigenetic and chromatin	NO	Increase in mtDNA and reduction in telomeric DNA were found contingent on the presence of Major Depression. No changes were reported in subjects who reported stressful life events, including CSA, but had never been depressed.
Mason et al., 2015	A	25–42 years	Blood	1135	Epigenetic and chromatin	NO	The relationship between physical abuse and telomere length seemed stronger than that for sexual abuse, but the effects were not statistically significant and there was no evidence of a dose-response relationship.
Vincent et al., 2017	A	20–84 years	Blood	180	Epigenetic and chromatin	NO	Only physical neglect in childhood activates telomere-shortening mechanisms (e.g., excessive cortisol, inflammatory activation), with the impact being greatest amongst older individuals, where the mechanism has had a longer time to evoke its detrimental effects.
Sosnowski et al., 2019	A	35–70 years	Blood	99	Epigenetic and chromatin	NO	There were no effects of exposure to abuse or abuse severity on mean telomere length. However, as social support increased, mean telomere length increased, but only for those women exposed to less severe forms of sexual abuse.
Warner et al., 2020	A	>18 years	Blood	3232	Epigenetic and chromatin	↓	Sexual abuse in childhood or adolescence was associated with a marker of accelerated biological aging, decreased telomere length. Sexual abuse was more strongly and consistently associated with telomere length than physical abuse.
Perroud et al., 2011	A	20–45 years	Blood	215	Epigenetic and chromatin	↑	Childhood sexual abuse was associated with increased NR3C1 promoter methylation. The severity of childhood abuses and neglect positively correlated with NR3C1 promoter methylation.
Beach et al., 2011	A	32–48 years	Blood	155	Epigenetic and chromatin	↑	CSA was associated with degree of methylation of the CpG island upstream of the SLC6A4, which mediated the impact of CSA on antisocial personality disorder symptoms, also potentiating the effect of 5HTT risk alleles.
York et al., 2013	A	39–57 years	Blood	51	Epigenetic and chromatin	↑	Female twins exposed to CSA exhibited a 1.63-fold average increase in the frequency of Micronuclei compared to non-exposed cotwins. The biological age of the CSA exposed twins was inferred to be 9.9 years older, suggesting cumulative effect.
**The transgenerational inheritance of CSA**							
Bosquet Enlow et al., 2018	A + C	Mother > 18 years; infants	Blood	151	Epigenetic and Chromatin	↓	Severity of CSA was associated with shorter cord blood relative telomere length (rTL), and greater maternal familial emotional support in childhood was associated with longer cord blood rTL among male infants.
Esteves et al., 2020	A + C	Mother > 18 years; child 4, 12, and 18 months	Buccal	155	Epigenetic and Chromatin	↓	Mothers’ high scores in the ACE questionnaire predicted shorter telomere length and emerging behavioral problems, especially externalizing ones in the next generation

*HPA, hypothalamic-pituitary-adrenal axis; BMI, body mass index; HIV, human immunodeficiency virus; IL-6, Interleukin 6; TNF-α, tumor necrosis factor- α; mtDNA, mitochondrial DNA; NR3C1, nuclear receptor subfamily 3 group C member 1; 5HTT, solute carrier family 6 member 4.*

One of the first human studies to report persistent changes in stress reactivity in adult survivors of early trauma showed that women with a history of childhood abuse, including sexual abuse, had greater pituitary adrenal and autonomic (plasma ACTH and cortisol) responses to stress compared with controls, in response to a standardized psychosocial laboratory stressor (Heim et al., 2000). Notably, this effect was particularly robust in women with current symptoms of depression and anxiety.

Schalinski et al. (2015) examined the impact of CSA on phasic and tonic HPA response in women with stress-related disorders who had experienced CSA by measuring, respectively, saliva cortisol and hair cortisol. Authors found a down regulation of saliva cortisol, measured at 4 different time points during an idiographic trauma interview (phasic-acute stress), in the CSA group versus controls. In contrast, the CSA group had higher hair cortisol levels compared with control, demonstrating increased levels of chronic stress in the CSA victims. To interpret this apparent paradox, the authors speculated that CSA dysregulates the HPA axis to be less reactive to acute stressors while delaying the activation, explaining the higher levels of cortisol in the hair.

A study by Bublitz and Stroud (2012) found that pregnant women with histories of CSA had significantly higher pre-pregnancy body mass index (BMI), greater anxiety symptoms, and increased saliva cortisol awakening responses (indicator frequently associated with psychological stress) during pregnancy than women with non-CSA histories and women who had never experienced abuse.

Finally, a pilot study suggests that altered cortisol secretion in individuals who suffered from CSA is a contributing factor to the risk of adult obesity (Hulme et al., 2015). Elevated glucocorticoid hormones in the CSA population might favor excess eating behaviors and negatively affect weight loss. This evidence is merely preliminary support for this hypothesis, necessitating further studies to understand the mechanisms that link CSA and adult obesity.

#### Immune System Dysfunction in Adults With History of Childhood Sexual Abuse

With the HPA axis, the immune system is likely to be a critical mediator of the greater risk for physical and mental illness, associated with childhood trauma.

A large body of literature has established that a traumatic childhood correlates with increased inflammatory markers, but when considering trauma subtypes, a clear association between CSA and inflammation is not always found.

Most studies have reported an increase in inflammatory activity – i.e., higher levels of IL-6, TNF-α, and C-reactive protein (Bertone-Johnson et al., 2012). However, researchers do not exclude the possibility that these alterations are secondary to other variables, such as psychiatric disorders, higher BMI, and unhealthy behaviors that frequently occur in victims of CSA.

Grosse et al. (2016) showed that in major depressive disorder (MDD) patients who had been traumatized at an early age, a higher severity of CSA was associated with greater levels of IL-6 and TNF-α. Neither cytokine was associated with more recent stressful experiences (past month and past 12 months), and the same effect was not found for the other types of abuse.

The link between CSA and adult HIV infection should also be highlighted. The rates of CSA in HIV-positive people are estimated to be 30 to 53% (McCall et al., 2018). In this population, HIV infection might be a consequence of behavioral and psychosocial factors, such as risky sexual behaviors. Nevertheless, the compromised immune function in these subjects might contribute to greater progression of AIDS (LeGrand et al., 2015).

Thus, disentangling the long-term consequences of CSA on the adult immune system functions is not easy. The complexity of this evaluation is well demonstrated in a recent systematic review that aimed to describe the association between CSA and indicators of immune activity. The diversity in studies that were collected, and the heterogeneity of CSA and trauma assessments prevented a meta-analysis from being performed (D’Elia et al., 2018).

Diverse could be the reasons why contrasting results are often observed in the context of immune system dysfunction. Several variables are commonly associated with CSA as well as with alterations on immune activity, representing important confounding factors. Above all, the comorbidity of CSA and psychiatric disorders in adult life is well established, and research clearly indicate that immune system alterations are present in several mental disorders. Moreover, among the population with history of CSA, the frequent increase in body mass index and unhealthy behaviors such as drug use, risky sex or smoking, all that elevate the risk of infection are further examples of potential confounders.

A second important factor may be intrinsic to the assessment of immune system functions. The immune activity throughout life is highly dynamic and responsive to diverse physiological and pathological conditions, such as the experience of stress, the gender (or the menstrual cycle phase that women are in at the time of assessment), the presence of infections. Thus, the timing when the adult assessment of cytokines level is performed is critical and several factors need to be controlled to help comparing different studies.

Eventually, the heterogeneity in the assessment of childhood traumatic experiences in different studies may also contribute to controversy in resulting effect of CSA on adult immune system dysfunctions. Indeed, CSA has a high prevalence of co-occurrence with multiple types of maltreatments, therefore considering sexual abuse as an isolate variable may be a confounding factor. Evidence demonstrates that when childhood maltreatment subtypes are studied separately, their impact on inflammatory markers is different (Baumeister et al., 2015). Moreover, victim’s age when abused are often not specified. Critical periods of plasticity are well defined during childhood development of brain and immune system, thus different time of exposure to CSA during development may result in very different consequences.

#### Altered DNA Methylation in Adults With a History of Childhood Sexual Abuse

Whereas epigenetic modifications, such as DNA methylation, have been studied widely as important mediators of traumatic childhood-induced adult pathologies, few studies have examined the effects of CSA on DNA methylation. Thus far, only promoters in candidate genes have been examined, including the genes for glucocorticoid receptor (*nr3c1*) and serotonin transporter (*slc6a4*). Epigenetic modification of the *nr3c1* gene might be a key factor of the long-term effects of CSA on HPA axis function.

Perroud et al. (2011) analyzed a wide group of patients who were diagnosed with borderline personality disorder (BPD), MDD, or PTSD and demonstrated that experiences with CSA and its severity, frequency, and number of type of maltreatments correlate positively with *nr3c1* methylation in the blood. This patter was especially evident in the subgroup of BPD subjects, in whom repeated abuse and CSA with penetration was associated with a higher percentage of methylation.

Beach et al. (2011) found similar results in the promoter region of the *slc6a4* gene, identifying methylation at 71 DNA CpG residues in a sample of lymphoblast cell lines that were derived from women from the Iowa adoptee study. The authors found that a higher degree of CpG island methylation upstream of the *slc6a4* gene was associated with reported CSA and symptoms of antisocial personality disorder (ASPD). Thus, CSA might contribute to greater methylation of this region, decreasing the responsiveness of the serotonergic system, in turn heightening aggressiveness, impairing impulse control, and engendering problematic social behavior – all of which collectively characterize ASPD.

#### Dysfunctional Cellular Processes in Adults With a History of Childhood Sexual Abuse

Following the hypothesis that CSA or a traumatic childhood in general affects developmental processes by accelerating cell division and aging, research has focused on determining quantitative indicators of these mechanisms in peripheral blood mononuclear cells. The most common parameters include the length of telomeres (measuring cellular aging); the level of mitochondrial DNA (mtDNA), which, when altered, indicate greater oxidative stress and inflammation; and the frequency of micronuclei (MN), (indicating chromosomal instability and altered cell division).

A large body of research on the effects of an adverse childhood has associated these experiences with shorter telomeres later in life. However, in CSA, the findings are often inconsistent.

Cai et al. (2015) analyzed a large sample of women to examine the association between stressful life events and mean telomere length and amount of mtDNA. Telomere length was significantly shorter and the amount of mtDNA was higher in MDD patients. Notably, both parameters correlated significantly in those who had experienced CSA or more stressful life events. The effect was cumulative, wherein the strength of the molecular markers increased with the severity of abuse. Notably, these changes were contingent on the presence of MDD. These results suggest that changes in the amount of mtDNA and telomere length are attributed to early life stress, even though they might be reversible and enter a depressed state in vulnerable individuals. One limitation of this study is that it did not investigate the specific effect of CSA on mtDNA and telomere length, instead, it evaluated the impact of CSA in combination with other stressful events.

On the contrary, Warner et al. (2020) specifically studied the effect of CSA and physical abuse on telomere length in adulthood. They observed that telomere length was 11.3% lower in individuals who experienced sexual abuse in childhood and adolescence, compared to controls. Notably, CSA resulted to be more associated with telomere length than physical abuse.

Nevertheless, inconsistent results have been reported by Sosnowski et al. (2019), who examined the association between exposure to CSA (and abuse severity) and mean telomere length in monozygotic female twins and determined whether social support moderated this correlate. Whereas no effect of abuse was found on mean telomere length, among women who experienced abuse in the forms of non-genital or genital contact without intercourse, higher level of social support was associated with longer mean telomere length. These findings have been explained assuming that individuals who experienced more severe forms of abuse have least capacity to form social ties and, thus, to benefit from the buffering effects of support throughout the lifespan.

Failures in identifying negative associations between telomere length and CSA are not uncommon (Mason et al., 2015; Vincent et al., 2017; Etzel et al., 2020). Telomere length depends highly on several factors, including age, parental age at birth, and life experiences. Thus, a limited sample size and high variability can often mask the effect of early traumas.

In the search for peripheral biomarkers of CSA-induced effects on chromosomal instability and aging, researchers have analyzed the frequency of MN in monozygotic twin pairs who are discordant for CSA (York et al., 2013). MN contain chromatin from structurally normal or abnormal chromosomes that are excluded from daughter cell nuclei during cell division. MN form due to a failure in migration, spontaneously or in response to the environment, ultimately altering cellular gene dosage. The frequency of MN increases with age and is elevated in patients with several serious health conditions (Bonassi et al., 2007; Murgia et al., 2007; Jones et al., 2011). York et al. (2013) have shown that the frequency of acquired somatic chromosomal changes, as measured by MN, varies by exposure to CSA, wherein victims of CSA have a 1.63-fold higher average increase in their frequency of MN compared with their twins. Moreover, this increase correlates positively with age only in CSA siblings; thus, their biological age was inferred to be on average 9.9 years older than their non-exposed twins.

### Child Sexual Abuse Scars: Transgenerational Inheritance

Growing evidence shows that traumas can be transmitted to offspring. Several studies have analyzed the transgenerational transmission of telomere length, estimating its hereditability at 70%. Shorter telomeres at birth might be linked to maternal oocyte telomere biology and maternal physiological stress responses in pregnancy. Recently, Esteves et al. (2020) found that higher level of maternal adverse childhood experiences predicted shorter telomere length in the next generation, which was not attributable to demographic factors, prenatal maternal stress score, or postnatal maternal depression. The scores also predicted emerging behavioral problems in infants, especially externalizing issues. However, the sample did not include sexually abused subjects exclusively.

Moreover, the inheritance of CSA-induced changes in maternal telomere length appears to be sex-dependent, primarily affecting male infants (Bosquet Enlow et al., 2018).

These results should encourage research to incorporate a multigenerational perspective in the study of the effects of early traumatic events.

## Discussion

Childhood sexual abuse has a substantial impact on psychological and organic aspects. Despite the large amount of evidence that clearly describes the immediate (on the child) and long-term (in adulthood) psychological outcomes of CSA, the neurobiological mechanisms that “translate” CSA to psychological changes are unknown. Further, few studies have determined whether and how the effects of CSA develop into structured medical conditions, such as immune and cardiovascular diseases, at later ages.

Research in this field has advanced thanks to the recent possibility of studying central nervous system pathophysiology in peripheral biospecimens, such as blood, saliva, urine, and tissues. Compared with central markers, peripheral markers are easily harvested and more amenable to banking; moreover, their acquisition is less invasive and inexpensive. Overall, the studies on peripherally detectable changes in this review indicate that CSA impacts physiological functions, including the HPA axis and immune system. At the cellular level, transcriptional regulation appears to be altered through epigenetic mechanisms, and high levels of oxidative stress and altered DNA telomerase activity indicate dysregulation of cellular processes.

We believe that the identification of short-term biological changes that are induced by CSA will allow pharmacological and psychological interventions to be implemented to prevent the development of structured psychiatric and medical illnesses in adulthood. Based on the individual variability in the vulnerability to CSA experiences, the identification of peripheral markers that predict the individual risk to later pathology would help the design of *ad hoc* treatments. Unfortunately, there is still a paucity of studies on this topic in children, necessitating greater effort in this area of research. It would be essential to design and promote longitudinal studies that investigate how CSA-induced biological changes modulate during development and eventually stabilize with growth. Furthermore, it would be essential to evaluate the association between these biological changes and psychological alterations. The evidence that neurobiological changes mediate the translation of a traumatic experience into psychopathology should be an essential issue to raise awareness about to encourage adherence to such studies.

The identification and validation of reliable peripheral biomarkers would aid in screens for CSA. Considering the emotional, psychological, and cultural aspects that prevent or hinder the victim from reporting sexual abuse (especially children, who have not fully developed the cognitive ability to recognize an act as abusive), it would be helpful to have peripheral changes that are recognized to be associated with (especially chronic) exposure to CSA. Moreover, CSA-specific biomarkers would be useful to distinguish it from other subtypes of child maltreatment and predict the type of psychological outcomes that result from this traumatic experience. Of course, this type of research in clinical contexts is quite difficult, due to the usual combination of early life traumatic experiences observable that produces confounding effects. Moreover, preclinical murine research in this case can hardly contribute, as a preclinical murine CSA model does not exist, and it is difficult to implement. Therefore, it would be desirable to enhance psychobiological research at the time CSA victims seek medical care (e.g., when these same victims access the emergency department) by identifying the immediate biological alterations resulting from this experience, monitoring these alterations over time, and assessing whether they correlate with the development of structural psychological alterations.

Finally, in the context of treatments for the resulting psychopathology, the existence of an association between CSA-induced changes and psychological symptoms implicates a reciprocal causal link. In such a case, we hypothesize that the modulation and variation of markers are associated with the degree of symptoms and that they reflect the success of the applied treatment.

Current research on this topic has several limitations. One major issue is the protocol that is used to record exposure to sexual abuse. Many studies differ in their method of assessing CSA, which complicates obtaining an organic collection of evidence on this topic. In addition, most studies measure exposure to CSA in adults through retrospective surveys, which can be misleading, because many events during life can alter the memory of abuse. Finally, an important limitation of these studies is that biological alterations have been investigated in a hypothesis-driven approach, whereas unbiased, genomewide studies would be better suited to shed light on novel biological pathways that are implicated in the translation of CSA to psychopathological risk.

## Conclusion

This review of current research demonstrates the need to characterize the effects of CSA to identify the mechanisms that mediate the structuring of psychological and medical illnesses and ultimately counteract them. Once identified, such peripheral biomarkers would also aid in the identification of more vulnerable individuals. Finally, these markers have the potential to be used in the clinical setting to monitor the remission of symptoms and treatment efficacy.

## Author Contributions

LL, CT, and VC made the bibliographic search and wrote the manuscript. All authors contributed to the article and approved the submitted version.

## Conflict of Interest

The authors declare that the research was conducted in the absence of any commercial or financial relationships that could be construed as a potential conflict of interest.

## Publisher’s Note

All claims expressed in this article are solely those of the authors and do not necessarily represent those of their affiliated organizations, or those of the publisher, the editors and the reviewers. Any product that may be evaluated in this article, or claim that may be made by its manufacturer, is not guaranteed or endorsed by the publisher.

## References

[B1] Aakvaag H. F. Thoresen S. Wentzel-Larsen T. Dyb G. (2017). Adult victimization in female survivors of childhood violence and abuse: the contribution of multiple types of violence. *Violence Against Women* 23 1601–1619. 10.1177/1077801216664427 27580984

[B2] Afifi T. O. MacMillan H. L. Boyle M. Taillieu T. Cheung K. Sareen J. (2014). Child abuse and mental disorders in Canada. *Can. Med. Assoc. J.* 186 E324–E332. 10.1503/cmaj.131792 24756625 PMC4050024

[B3] Assink M. Spruit A. Schuts M. Lindauer R. van der Put C. E. Stams G. J. J. (2018). The intergenerational transmission of child maltreatment: a three-level meta-analysis. *Child Abuse Negl.* 84 131–145. 10.1016/j.chiabu.2018.07.037 30086419

[B4] Atabay E. Arman A. R. (2019). Oxidative stress in children with sexual abuse may be elevated and correlate with history of psychiatric treatment: a cross-sectional case-control study. *Psychiatry Behav. Sci.* 9 102–111. 10.5455/PBS.20181128074618

[B5] Baumeister D. Akhtar R. Ciufolini S. Pariante C. M. Mondelli V. (2015). Childhood trauma and adulthood inflammation: a meta-analysis of peripheral C-reactive protein, Interleukin-6 and tumour necrosis factor-α. *Mol. Psychiatry* 21 642–649. 10.1038/mp.2015.67 26033244 PMC4564950

[B6] Beach S. R. Brody G. H. Todorov A. A. Gunter T. D. Philibert R. A. (2011). Methylation at 5HTT mediates the impact of child sex abuse on women’s antisocial behavior: an examination of the Iowa adoptee sample. *Psychosom. Med.* 73 83–87. 10.1097/PSY.0b013e3181fdd074 20947778 PMC3016449

[B7] Bertone-Johnson E. R. Whitcomb B. W. Missmer S. A. Karlson E. W. Rich-Edwards J. W. (2012). Inflammation and early-life abuse in women. *Am. J. Prev. Med.* 43 611–620. 10.1016/j.amepre.2012.08.014 23159256 PMC3504353

[B8] Bonassi S. Znaor A. Ceppi M. Lando C. Chang W. P. Holland N. (2007). An increased micronucleus frequency in peripheral blood lymphocytes predicts the risk of cancer in humans. *Carcinogenesis* 28 625–631. 10.1093/carcin/bgl177 16973674

[B9] Bosquet Enlow M. Bollati V. Sideridis G. Flom J. D. Hoxha M. Hacker M. R. (2018). Sex differences in effects of maternal risk and protective factors in childhood and pregnancy on newborn telomere length. *Psychoneuroendocrinology* 95 74–85. 10.1016/j.psyneuen.2018.05.025 29803183 PMC6109592

[B10] Bublitz M. H. Stroud L. R. (2012). Childhood sexual abuse is associated with cortisol awakening response over pregnancy: preliminary findings. *Psychoneuroendocrinology* 37 1425–1430. 10.1016/j.psyneuen.2012.01.009 22341730 PMC3368071

[B11] Cai N. Chang S. Li Y. Li Q. Hu J. Liang J. (2015). Molecular signatures of major depression. *Curr. Biol.* 25 1146–1156. 10.1016/j.cub.2015.03.008 25913401 PMC4425463

[B12] Center for Disease Control and Prevention (2021). *Preventing Child Sexual Abuse.* Available online at: https://www.cdc.gov/violenceprevention/childabuseandneglect/childsexualabuse.html (accessed September 1, 2021).

[B13] Clayton E. Jones C. Brown J. Taylor J. (2018). The aetiology of child sexual abuse: a critical review of the empirical evidence. *Child Abuse Rev.* 27 181–197. 10.1002/car.2517

[B14] Cyr C. Euser E. M. Bakermans-Kranenburg M. J. Van Ijzendoorn M. H. (2010). Attachment security and disorganization in maltreating and high-risk families: a series of meta-analyses. *Dev. Psychopathol.* 22 87–108. 10.1017/S0954579409990289 20102649

[B15] D’Elia A. Matsuzaka C. T. Neto J. Mello M. F. Juruena M. F. Mello A. F. (2018). Childhood sexual abuse and indicators of immune activity: a systematic review. *Front. Psychiatry* 9:354. 10.3389/fpsyt.2018.00354 30127754 PMC6088139

[B16] Devries K. M. Mak J. Y. Child J. C. Falder G. Bacchus L. J. Astbury J. (2014). Childhood sexual abuse and suicidal behavior: a meta-analysis. *Pediatrics* 133 e1331–e1344. 10.1542/peds.2013-2166 24733879

[B17] Esteves K. C. Jones C. W. Wade M. Callerame K. Smith A. K. Theall K. P. (2020). Adverse childhood experiences: implications for offspring telomere length and psychopathology. *Am. J. Psychiatry* 177 47–57. 10.1176/appi.ajp.2019.18030335 31509004 PMC7273739

[B18] Etzel L. Hastings W. J. Mattern B. C. Oxford M. L. Heim C. Putnam F. W. (2020). Intergenerational transmission of childhood trauma? Testing cellular aging in mothers exposed to sexual abuse and their children. *Psychoneuroendocrinology* 120:104781. 10.1016/j.psyneuen.2020.104781 32629221 PMC7502488

[B19] Ford J. D. Delker B. C. (2018). Polyvictimization in childhood and its adverse impacts across the lifespan: introduction to the special issue. *J. Trauma Dissociation* 19 275–288. 10.1080/15299732.2018.1440479 29547074

[B20] Goodman M. L. Hindman A. Keiser P. H. Gitari S. Ackerman Porter K. Raimer B. G. (2020). Neglect, sexual abuse, and witnessing intimate partner violence during childhood predicts later life violent attitudes against children among Kenyan women: evidence of intergenerational risk transmission from cross-sectional data. *J. Interpers. Violence* 35 623–645. 10.1177/0886260516689777 29294640

[B21] Grosse L. Ambrée O. Jörgens S. Jawahar M. C. Singhal G. Stacey D. (2016). Cytokine levels in major depression are related to childhood trauma but not to recent stressors. *Psychoneuroendocrinology* 73 24–31. 10.1016/j.psyneuen.2016.07.205 27448525

[B22] Hailes H. P. Yu R. Danese A. Fazel S. (2019). Long-term outcomes of childhood sexual abuse: an umbrella review. *Lancet Psychiatry* 6 830–839. 10.1016/S2215-0366(19)30286-X 31519507 PMC7015702

[B23] Halpern S. C. Schuch F. B. Scherer J. N. Sordi A. O. Pachado M. Dalbosco C. (2018). Child maltreatment and illicit substance abuse: a systematic review and meta-analysis of longitudinal studies. *Child Abuse Rev.* 27 344–360. 10.1002/car.2534

[B24] Heim C. Newport D. J. Heit S. Graham Y. P. Wilcox M. Bonsall R. (2000). Pituitary-adrenal and autonomic responses to stress in women after sexual and physical abuse in childhood. *JAMA* 284 592–597. 10.1001/jama.284.5.592 10918705

[B25] Hulme P. A. McBride C. L. Kupzyk K. A. French J. A. (2015). Pilot study on childhood sexual abuse, diurnal cortisol secretion, and weight loss in bariatric surgery patients. *J. Child Sex. Abuse* 24 385–400. 10.1080/10538712.2015.1022293 26061023 PMC5996386

[B26] Jones K. H. York T. P. Juusola J. Ferreira-Gonzalez A. Maes H. H. Jackson-Cook C. (2011). Genetic and environmental influences on spontaneous micronuclei frequencies in children and adults: a twin study. *Mutagenesis* 26 745–752. 10.1093/mutage/ger042 21765037 PMC3198889

[B27] Langevin R. Hébert M. Allard-Dansereau C. Bernard-Bonnin A. C. (2016). Emotion regulation in sexually abused preschoolers: the contribution of parental factors. *J. Trauma Stress* 29 180–184. 10.1002/jts.22082 26915665

[B28] LeGrand S. Reif S. Sullivan K. Murray K. Barlow M. L. Whetten K. (2015). A review of recent literature on trauma among individuals living with HIV. *Curr. HIV/AIDS Rep.* 12 397–405. 10.1007/s11904-015-0288-2 26419376 PMC4837695

[B29] Madigan S. Bakermans-Kranenburg M. J. Van Ijzendoorn M. H. Moran G. Pederson D. R. Benoit D. (2006). Unresolved states of mind, anomalous parental behavior, and disorganized attachment: a review and meta-analysis of a transmission gap. *Attach. Hum. Dev.* 8 89–111. 10.1080/14616730600774458 16818417

[B30] Mason S. M. Prescott J. Tworoger S. S. DeVivo I. Rich-Edwards J. W. (2015). Childhood physical and sexual abuse history and leukocyte telomere length among women in middle adulthood. *PLoS One* 10:e0124493. 10.1371/journal.pone.0124493 26053088 PMC4459951

[B31] McCall J. Lauridsen-Hoegh P. Unger D. Phillips J. C. Kille J. (2018). Childhood sexual abuse in a population of patients living with HIV: prevalence and impact. *J. Assoc. Nurses AIDS Care* 29 466–474. 10.1016/j.jana.2017.12.001 29306671

[B32] Muller D. Errington S. Szabo C. P. Pitts N. Jacklin L. (2014). Cortisol and IL-6 responses to stress in female children presenting at a sexual abuse clinic. *J. Child Adolesc. Trauma* 7 185–191. 10.1007/s40653-014-0019-7

[B33] Murgia E. Maggini V. Barale R. Rossi A. M. (2007). Micronuclei, genetic polymorphisms and cardiovascular disease mortality in a nested case-control study in Italy. *Mutat. Res.* 621 113–118. 10.1016/j.mrfmmm.2007.02.015 17448506

[B34] Perroud N. Paoloni-Giacobino A. Prada P. Olié E. Salzmann A. Nicastro R. (2011). Increased methylation of glucocorticoid receptor gene (NR3C1) in adults with a history of childhood maltreatment: a link with the severity and type of trauma. *Transl. Psychiatry* 1:e59. 10.1038/tp.2011.60 22832351 PMC3309499

[B35] Putnam F. W. (2003). Ten-year research update review: child sexual abuse. *J. Am. Acad. Child Adolesc. Psychiatry* 42 269–278. 10.1097/00004583-200303000-00006 12595779

[B36] Putnam K. T. Harris W. W. Putnam F. W. (2013). Synergistic childhood adversities and complex adult psychopathology. *J. Trauma Stress* 26 435–442. 10.1002/jts.21833 23893545

[B37] Ridout K. K. Levandowski M. Ridout S. J. Gantz L. Goonan K. Palermo D. (2018). Early life adversity and telomere length: a meta-analysis. *Mol. Psychiatry* 23 858–871. 10.1038/mp.2017.26 28322278 PMC5608639

[B38] Saywitz K. J. Mannarino A. P. Berliner L. Cohen J. A. (2000). Treatment of sexually abused children and adolescents. *Am. Psychol.* 55 1040–1049. 10.1037/0003-066X.55.9.1040 11036707

[B39] Schalinski I. Elbert T. Steudte-Schmiedgen S. Kirschbaum C. (2015). The cortisol paradox of trauma-related disorders: lower phasic responses but higher tonic levels of cortisol are associated with sexual abuse in childhood. *PLoS One* 10:e0136921. 10.1371/journal.pone.0136921 26317554 PMC4552952

[B40] Shalev I. Moffitt T. E. Sugden K. Williams B. Houts R. M. Danese A. (2013). Exposure to violence during childhood is associated with telomere erosion from 5 to 10 years of age: a longitudinal study. *Mol. Psychiatry* 18 576–581. 10.1038/mp.2012.32 22525489 PMC3616159

[B41] Sigurdardottir S. Halldorsdottir S. (2018). Screaming body and silent healthcare providers: a case study with a childhood sexual abuse survivor. *Int. J. Environ. Res. Public Health* 15:94. 10.3390/ijerph15010094 29316709 PMC5800193

[B42] Şimşek Ş Kaplan İ Uysal C. Yüksel T. Alaca R. (2016). The levels of cortisol, oxidative stress, and DNA damage in the victims of childhood sexual abuse: a preliminary study. *J. Child Sex. Abuse* 25 175–184. 10.1080/10538712.2016.1123790 26934543

[B43] Slep A. M. S. Heyman R. E. Foran H. M. (2015). Child maltreatment in DSM-5 and ICD-11. *Fam. Process* 54 17–32. 10.1111/famp.12131 25615555

[B44] Sosnowski D. W. Kliewer W. York T. P. Amstadter A. B. Jackson-Cook C. K. Winter M. A. (2019). Familial support following childhood sexual abuse is associated with longer telomere length in adult females. *J. Behav. Med.* 42 911–923. 10.1007/s10865-019-00014-7 30671916 PMC7703807

[B45] Vincent J. Hovatta I. Frissa S. Goodwin L. Hotopf M. Hatch S. L. (2017). Assessing the contributions of childhood maltreatment subtypes and depression case-control status on telomere length reveals a specific role of physical neglect. *J. Affect. Disord.* 213 16–22. 10.1016/j.jad.2017.01.031 28187293 PMC6191534

[B46] Warner E. T. Zhang Y. Gu Y. Taporoski T. P. Pereira A. DeVivo I. (2020). Physical and sexual abuse in childhood and adolescence and leukocyte telomere length: a pooled analysis of the study on psychosocial stress, spirituality, and health. *PLoS One* 15:e0241363. 10.1371/journal.pone.0241363 33125425 PMC7598522

[B47] York T. P. Brumelle J. Juusola J. Kendler K. S. Eaves L. J. Amstadter A. B. (2013). Increased frequency of micronuclei in adults with a history of childhood sexual abuse: a discordant monozygotic twin Study. *PLoS One* 8:e55337. 10.1371/journal.pone.0055337 23383158 PMC3559336

